# Controllable preparation of an ice cream-shaped hollow sphere array†

**DOI:** 10.1039/d2ra00236a

**Published:** 2022-03-22

**Authors:** Yang Liu, Xinlong Sun, Feng Zhao, Fei Zhan, Bo Zhang, Jun-Heng Fu, Lei Wang, Jing Liu

**Affiliations:** Beijing Key Lab of Cryo-biomedical Engineering and Key Lab of Cryogenics, Technical Institute of Physics and Chemistry, Chinese Academy of Sciences Beijing 100190 P. R. China leiwang@mail.ipc.ac.cn; Specialized Robot Engineering and Technological Centre of Hainan Province, Hainan Vocational University of Science and Technology Haikou 571126 China; School of Electrical and Electronic Engineering, Shijiazhuang Tiedao University Hebei 050043 China; Institute of Advanced Structure Technology, Beijing Key Laboratory of Lightweight Multi-Functional Composite Materials and Structures, Beijing Institute of Technology Beijing 100081 China; Department of Biomedical Engineering, School of Medicine Tsinghua University Beijing 100084 PR China

## Abstract

Hollow microspheres with high specific surface area are widely used in thermal insulation, drug delivery and sustained release, catalysis and optical absorption. Eutectic gallium–indium (EGaIn) undergoes phase transformation and oxidation when heated in aqueous solution, which can provide a crystal seed and preferential growth environment for nanomaterials. Therefore, it is very promising to further study the application of liquid metal in functional and structural nanomaterials. In this study, a EGaIn-based ice cream-shaped hollow sphere array with nanostructures was firstly synthesized on the designed hole array model using a hydrothermal process, and then the surface was further modified by fluorination to form a superhydrophobic film. Different sizes of the hollow Eutectic gallium–indium zinc oxide (EGaIn-ZnO) microspheres could be easily achieved by varying the size of the model, hence leading to controllable wettability. Furthermore, hollow microspheres hold much air, making it feasible for application in the field of anti-ice and thermal insulation.

## Abstract 2

In a hydrothermal environment, EGaIn liquid droplets in hole arrays transfer from liquid state into solid hollow spheres. They hold much air between the solid and liquid interface, enhancing the thermal insulation and superhydrophobic performance.

## Graphical abstract

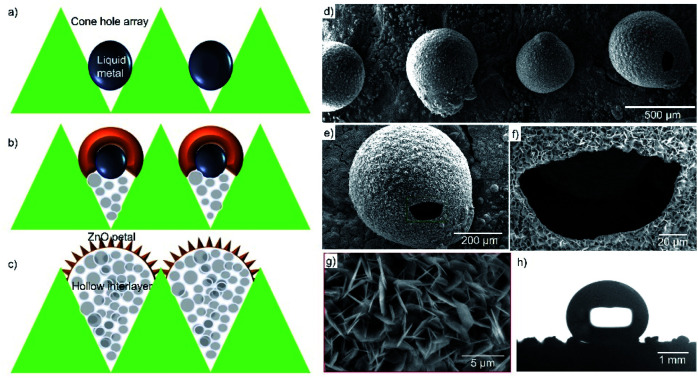

## 1 Introduction

Nanoscale building blocks have been of great interest in recent decades, which is due not only to the enhancement of the understanding for self-assembled nano units but also the prosperous application potential in catalysis, gas sensors and battery materials. Huge efforts have been done on investigating the hollow microsphere structures which could largely enhance the development of superhydrophobic coatings and sensor properties.^1–3^ Hierarchically porous polymer microspheres prepared by non solvent assisted electrospraying paves the way for fabricating superhydrophobic coatings.^4^ Hedgehog-shaped hollow vanadium pentoxide (V_2_O_5_) microspheres with nanorods were synthesized by a mediated polyol process.^5^ Treating ethylene glycol (EG) with polyvinylpyrrolidone (PVP) in the presence of metal salt is a widely used method to assemble hollow microspheres with nanorods on the surface. Due to the large band gap (3.37 eV) of zinc oxide (ZnO),^6^ it has attracted more attention on the preparation and application of ZnO nano building blocks later. ZnO nanorods could be converted to hollow microhemispheres through solvothermal method in the presence of zinc and ethylene glycol.^7,8^ Several other ways to synthesize ZnO hollow microspheres had been pointed out recently, such as templating by carbon microspheres,^9^ hydrothermal synthesis by spherobacterium,^10^ base-erosion mechanism through applying tetraamminezic(ii) ion (Zn(NH_3_)_4_^2+^) precursor in ethanol solvent,^11^ and preparation by monodisperse positively charged polymer particles.^12^ These fabricated self-assemble ZnO microspheres opened the way in gas detections, given their extreme sensitivity to ethanol and ammonia. It is urgent to obtain hollow spheres and nanostructures simultaneously through a single experiment, which can not only reduce the cost but also save the experiment time. In this paper, a novel facile hydrothermal process applying methenamine and zinc nitrate to assemble hollow petal-like EGaIn-ZnO microspheres was proposed. And the hollow structures then were examined with EDS and SEM approachs, which provided comprehensive assessments for the formation of microspheres. This facile strategy of the fabricated hollow ZnO microspheres under the help of liquid metal shows a more promising future for the application in catalysis, gas sensors and battery materials.

## 2 Experimental

### 2.1 Preparation of superhydrophobic EGaIn film

Firstly, the EGaIn was rubbed into the as-prepared pores homogenously, and the excess EGaIn residue left on the surface of substrate was wiped off. The hydrothermal reaction solution for the fabrication of hollow EGaIn-ZnO microspheres was prepared from an aqueous solution containing 2.50 mmol methenamine, 4.58 mmol zinc nitrate in 100 mL deionized water. When a clear solution was formed, the substrate with an inclined angle >90°, was then placed into a 100 mL stainless steel autoclave. After being sealed, the autoclave was heated at 95 °C for 10 h. The prepared film was placed in a desiccator with fluorosilane for further treatment.

### 2.2 Characterization techniques

The surface morphology of petal-like structures was investigated using a scanning electron microscope (SEM). The chemical composition was characterized using Energy Dispersive Spectroscopy (EDS). The wetting behavior of droplets on the surface of prepared sample was testing by the contact angle measurement instrument (JC200d3) at room temperature.

## 3 Results and discussion

### 3.1 Analysis of the prepared superhydrophobic surface

**Fig. 1:**
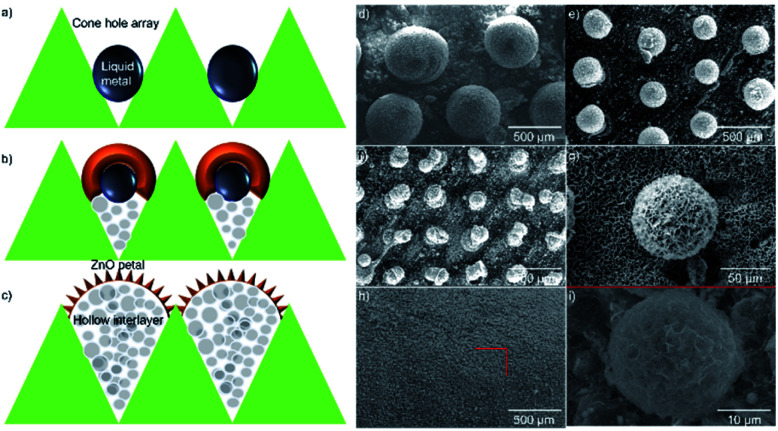
(a–c) The liquid–solid transformation of EGaIn liquid droplets in the holes at hydrothermal environment. (d–i) SEM images of the top view of microspheres. The hierarchical growth of spheres could be achieved by varying the size of the pores.

For schematic illustration, the substrate, made from the surface with apophysis, is where microspheres could be formed in the holes. The prepared substrate could fabricate microspheres with a diameter of 20 μm (Fig. S1†). In the hydrothermal environment, the liquid metal placed in the designed substrate would move upwards due to the capillary effect.^13,14^ The formation of a dense oxide film on its surface prevents further oxidation since gallium is easily oxidized in the presence of oxygen. When the liquid droplet reaches the equilibrium position, the ZnO layer, encasing EGaIn liquid metal, would react with the aqueous solution to start constructing petal-like nanostructures on the surface. At this point, small portions of EGaIn-ZnO microspheres would turn into porous structure. When the reaction proceeds, the EGaIn-ZnO microspheres could complete the transition from liquid to solid metal forming a hollow interlayer inside (Fig. 1a–c) The morphology of nanostructures on the surface was evaluated using SEM. Fig. 1d–i show the overall views of the prepared microspheres with various sizes. The size of microspheres is controlled by two factors: the size of the pore and the amount of liquid metal in the pore. Excess liquid metal would result in the internal liquid metal crowding out. However, when there is an inadequate amount of the EGaIn in the hole, the liquid metal droplet would remain in the cone. But the nanostructures could still be formed (Fig. S2†).

For the fabrication of nanostructures, methenamine and zinc nitrate are added directly to the system before the hydrothermal reaction proceeds. Methenamine would establish a wealthy basic environment for nucleation and crystal growth. Zinc nitrate is acting as the dispersant while providing Zn^2+^ which is essential for the formation of ZnO and Zinc hydroxide (ZnOH) under basic conditions. During the fabrication of petal-like nanostructures, the oxidized outer layer of EGaIn would provide the crystal seed for the system. The growth of primary ZnO nanoparticles which were formed from ZnO seeds, was inhibited by zinc nitrate to construct stable dispersibility. On the basis of polarity, the oxygen atom in ZnO would be attracted to the amine group, hence forming a new polar molecule. This process is achieved with minimum particle surface energy due to the strong driving force between neighboring nanoparticles.^15,16^ These new polar chains would aggregate with each other to assemble the petal-like structure on the microspheres (Fig. S3†) during the annealing process. If there is inadequate amount of EGaIn in the pore to construct the microsphere, ZnO shell would not be closed (Fig. S4 and S5†). In contrast, when there is excess EGaIn to start with, the volume of EGaIn-ZnO microspheres would keep increasing after the shell is closed. As the result, excess EGaIn could react with aqueous solution and be expelled (Fig. S6†).

**Fig. 2:**
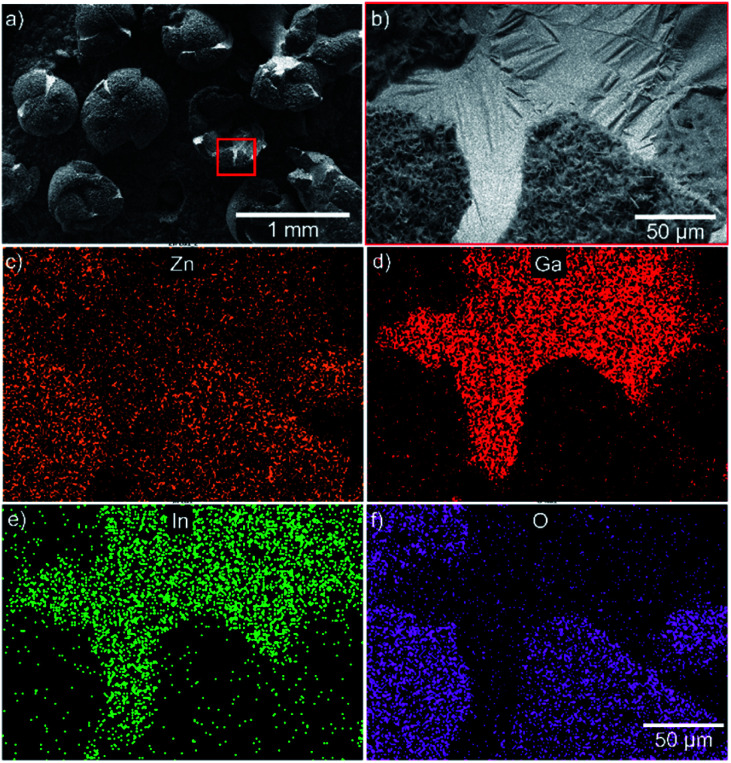
(a and b) SEM images of the broken spheres show that EGaIn-ZnO microspheres are hollow. (c–f) EDS analysis of Zn, Ga, In and O.

The elemental distribution mapping of the surface was characterized through EDS. Fig. 2 shows that the surface of the EGaIn-ZnO microspheres is mainly composed of Zn and O, which is also the composition of nanostructures. The exposed inner layer of hollow EgaIn-ZnO microsphere is mainly constituted by Ga and In, which is the element presented in the liquid metal. That proves the liquid–solid transformation during the fabrication of nano particles with petal-like structures.

**Fig. 3:**
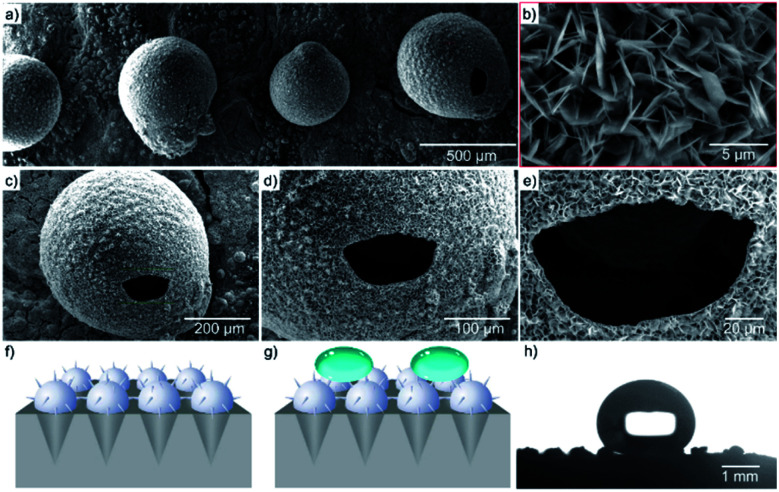
(a–e) The modified view of the microspheres. (f and g) The schematic illustration when water droplet was added to the prepared film. (h) The still view captured by SurfaSpector, and the measured contact angle is 150°. While, the contact angle is less than 10° before fluorination.

Small polar solvent, water, was used in this experiment so that the reversible hydrogen bonding could occur. This is because the large and polar solvents could directly link the nanoparticles in an irreversible direction to form irregular microsphere. Hah *et al.*^17^ had pointed out that long chain solvent introduced to the system would reduce the polarity and hence affects the growth of seeds. The formation process of the nanostructures on the surface of EGaIn microsphere has not been studied intensively. However, there were several explanations for the construction of ZnO nanoparticles, which have the same petal-like structure. Liu *et al.*^18^ proposed an explanation for the construction of hollow ZnO spheres with the use of EDA in polar media. The formation of the petal-like nanostructures on the surface is through assembling process, not layer by layer formation. In Fig. 3(a–e), it could be observed that the microsphere encased through open ring. The liquid metal inside tended to inflate and transform into hollow structure in hydrothermal environment. Fig. S4† showcased the wall of the hollow structure is approximately 5 μm. Zhu *et al.*^19^ pointed out a template-free hydrothermal method to synthesize ZnO hollow microspheres at low temperature. A high concentration of trisodium citrate was used in the reaction in order to control the growth of seeds and the size of the nanostructures formed at the end. This simple synthesis process with efficient reaction time and high yield, could be the candidate in the large industrial production. Fig. 3(f–h) displays the schematic view when a water droplet was added to the prepared EGaIn film. The petal-like structures on the surface of microspheres would prevent the droplet attaching the surface, consequently achieving superhydrophobicity. Also, it is worthwhile to point out that the large volume of air between the solid and liquid interface significantly increases the thermal insulating performance and anti-icing behavior. At −2 °C (in refrigerator), the water droplet could freeze on the surface of EGaIn-ZnO microspheres after 5.1 min. While on a PVC surface, it takes only 0.2 min to freeze, and the water droplet tends to spread out (Fig. S7†). The experimental results indicate that these EGa-ZnO hollow microspheres with petal-like nanostructure could provide a promising future for the potential application in anti-icing and thermal isolation field.

## 4 Conclusions

Hierarchical hollow petal-like ZnO-EGaIn microspheres were successfully fabricated by the hydrothermal process. The formation of the microspheres with petal-like structure is through assembling process which allows the surface to be filled. The surface of the EGaIn-ZnO hollow microspheres are mainly composed of Zn and O element, and the inner compositions are Ga and In. This had proven that the liquid metal encased by the ZnO layer tend to inflate and form porous structure under hydrothermal conditions. Given that the applicable surface area (1.8 m^2^ g^−1^) possessed by microspheres, they might be applied in the area of the thermal isolation, catalysis and adsorptive removal of organic wastewater pollutants.

## Author contributions

Lei Wang supervised this work, performed the experiment. Yang Liu and Lei Wang wrote the manuscript. Yang Liu, Xinlong Sun, Fei Zhan and Jun-Heng Fu designed the experiment. Feng Zhao, Bo Zhang and Jing Liu provided theoretical support for this study.

## Conflicts of interest

The authors declare no competing financial interests.

## Supplementary Material

RA-012-D2RA00236A-s001
