# Supplementary material for: Controllable preparation of an ice cream-shaped hollow sphere array
Source: RSC Adv. 2022 Mar 22;12(15):8936–9. doi: 10.1039/d2ra00236a (PMC8985163; doi:10.1039/d2ra00236a)
Supplement: RA-012-D2RA00236A-s001 [file RA-012-D2RA00236A-s001.pdf]

Electronic Supplementary Material (ESI) for RSC Advances.
This journal is © The Royal Society of Chemistry 2022

**Controllable Preparation of Ice Cream-Shaped Hollow Sphere Array**

Yang Liu [a)], Xinlong Sun [a)], Feng Zhao [b)], Fei Zhan [c)], Bo Zhang [d)], Jun-Heng Fu [a)], Lei Wang [a)]*, Jing Liu [a, e)]

a) Beijing Key Lab of Cryo-biomedical Engineering and Key Lab of Cryogenics, Technical Institute of Physics and Chemistry, Chinese Academy of Sciences, Beijing 100190, P. R. China

b) Specialized Robot Engineering and Technological Centre of Hainan Province, Hainan Vocational University of Science and Technology, Haikou 571126, China

c) School of Electrical and Electronic Engineering, Shijiazhuang Tiedao University, Hebei 050043, China

d) Institute of Advanced Structure Technology，Beijing Key Laboratory of Lightweight Multi-Functional Composite Materials and Structures, Beijing Institute of Technology, Beijing 100081, China

e) Department of Biomedical Engineering, School of Medicine Tsinghua University, Beijing 100084, PR China

*Correspondence to: leiwang@mail.ipc.ac.cn

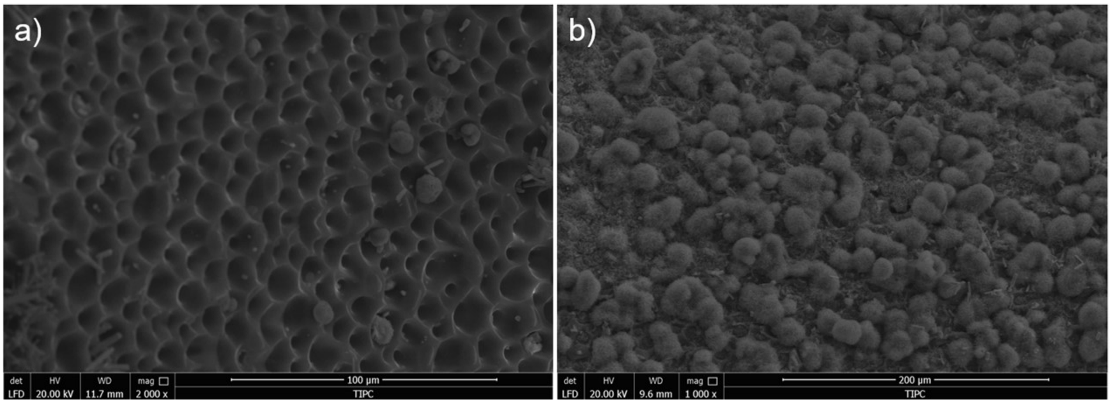


Figure S1. SEM images of (a) the substrate, made from the surface with apophysis, where microspheres could be formed in the holes. (b) the prepared microspheres with a diameter of 20 micrometers.

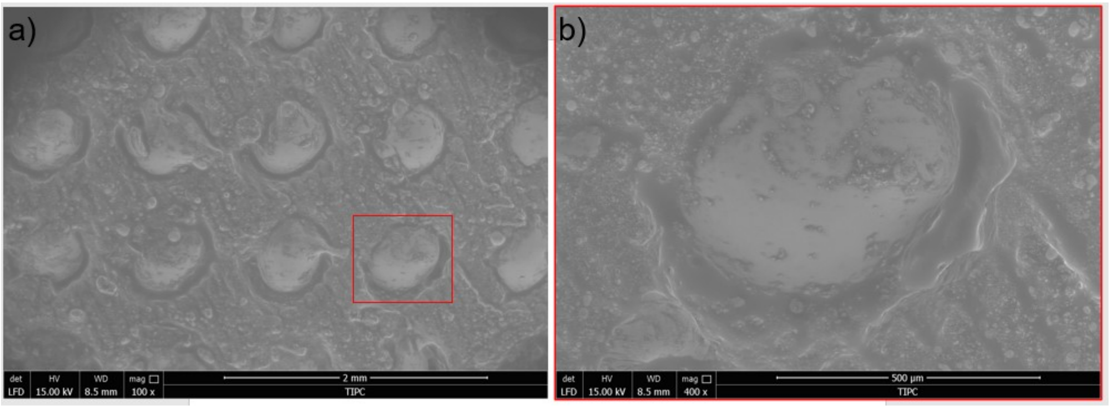


Fig. S2. Insufficient amount of liquid metal in the hole, but the liquid to solid transformation can still occur.

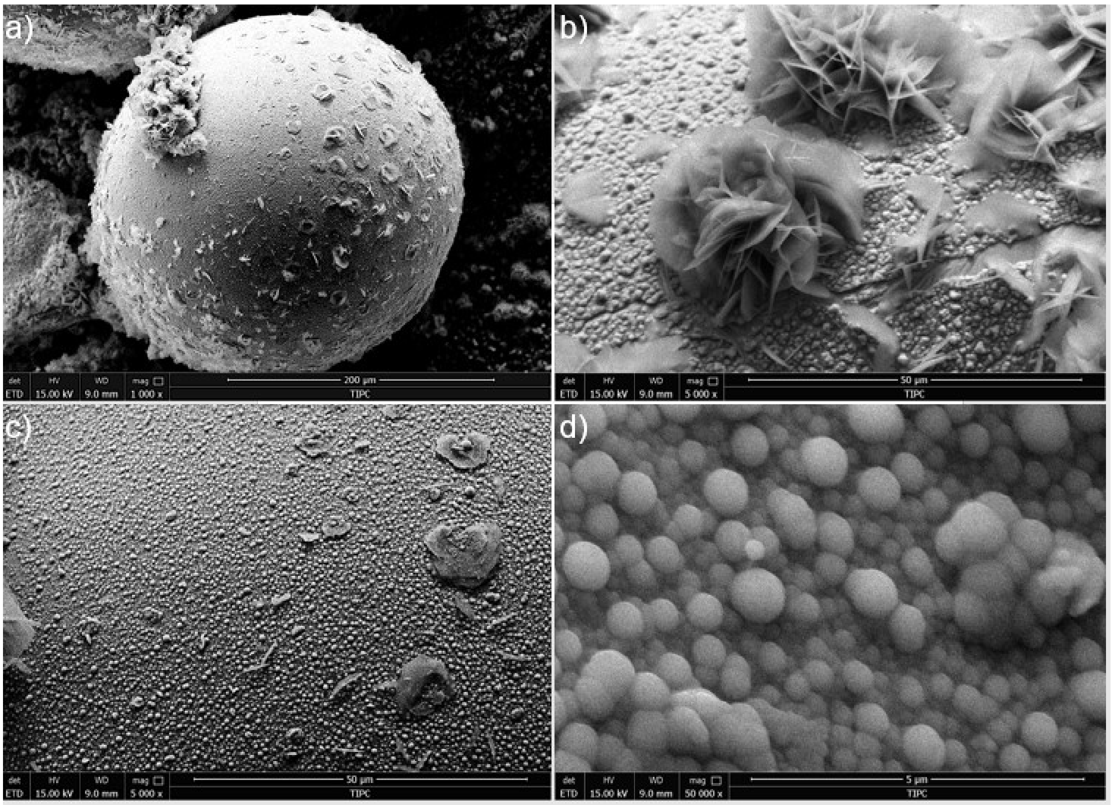


Fig. S3. The formation process of nanostructures under SEM investigation. The spherical seed crystal was produced at first and followed by the construction of petal-like structures.

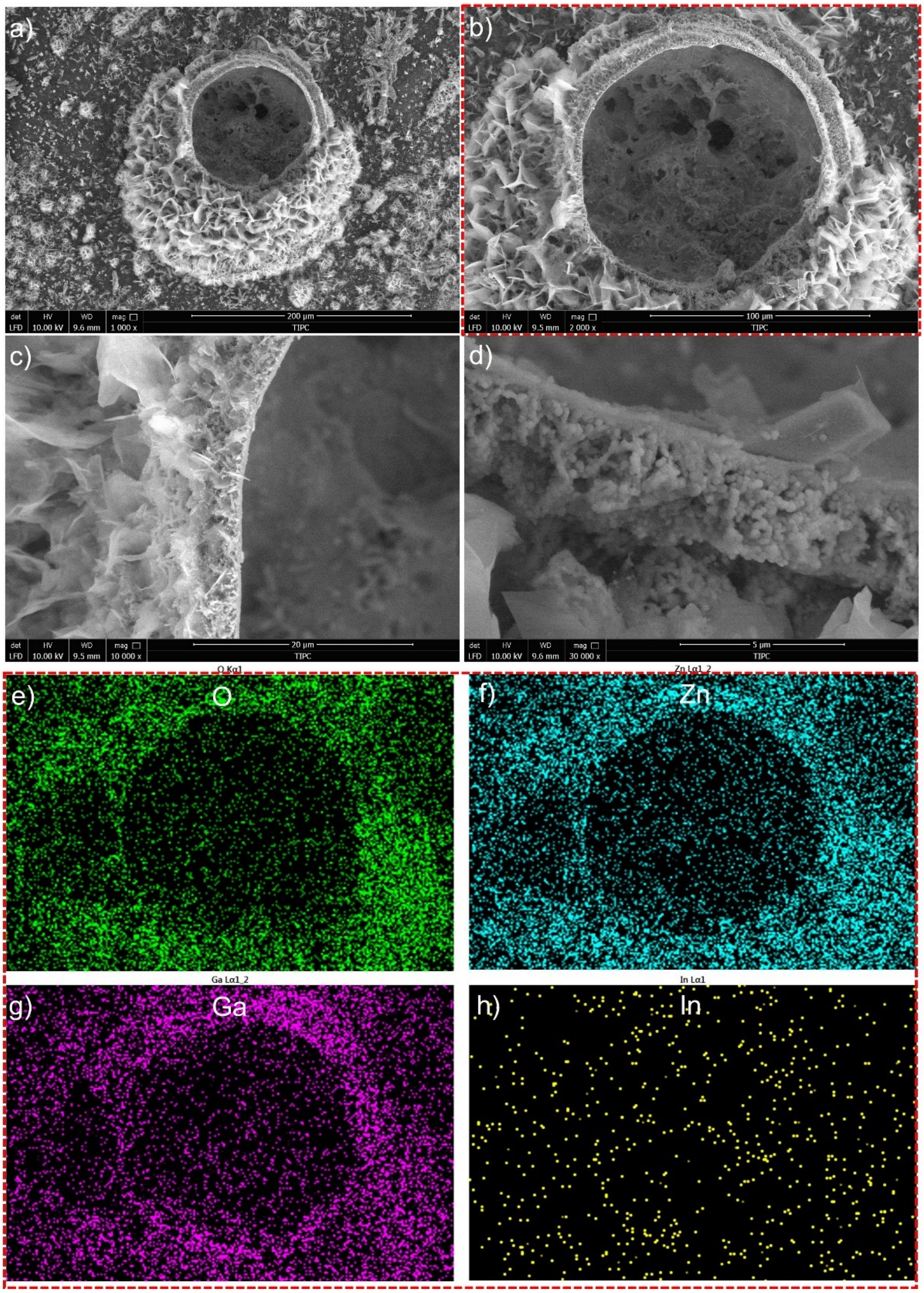


Fig. S4. SEM and EDS images of the cross section of hollow microspheres, which shows the thickness of the wall is 5 micrometers.

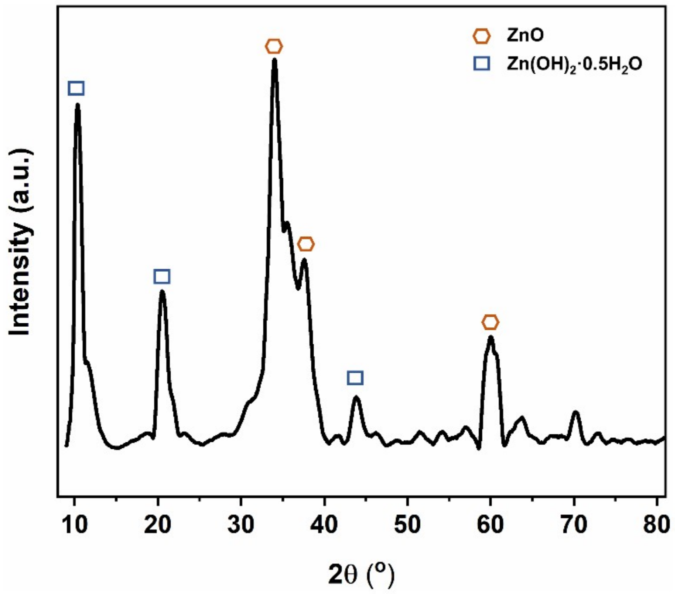


Fig. S5. X-Ray Diffraction (XRD) test of the hollow sphere. It is composed by ZnO and $Zn(OH)_2 0.5H_2O$.

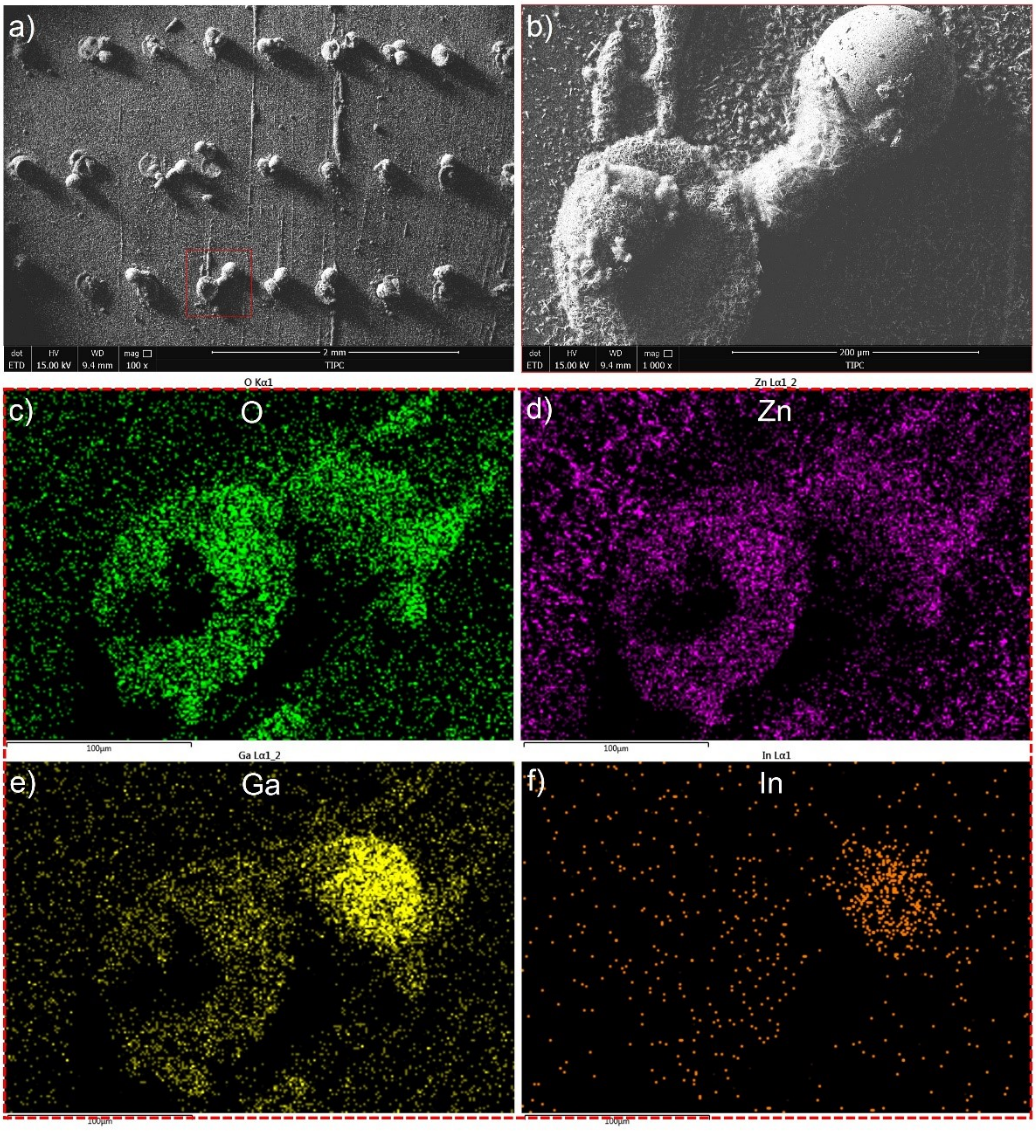


Fig. S6. SEM and EDS images of the cross section of hollow microspheres, which shows the excess EGaIn was expelled.

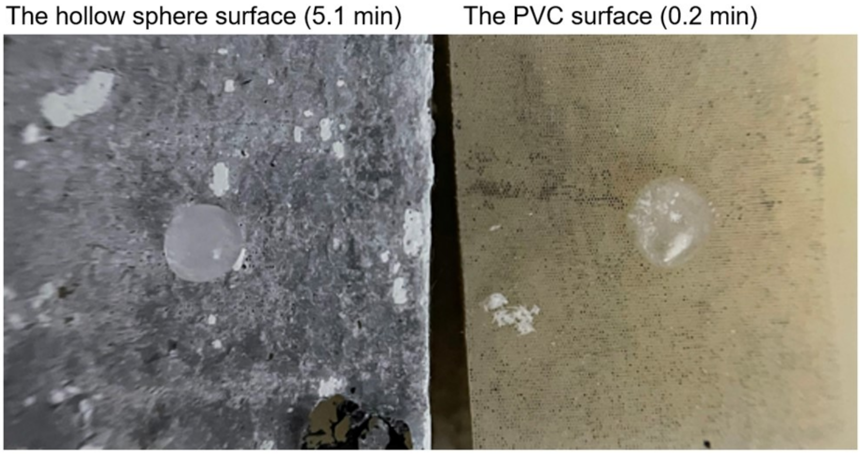

Fig. S7. The anti-icing performance. The droplets freeze on the hollow sphere surface and PVC surface is 5.1 min and 0.2 min, respectively.
